# Supplementary material for: End Sequence Analysis Toolkit (ESAT) expands the extractable information from single-cell RNA-seq data
Source: Genome Res. 2016 Oct;26(10):1397–410. doi: 10.1101/gr.207902.116 (PMC5052061; doi:10.1101/gr.207902.116)
Supplement: Supplemental Material [file supp_gr.207902.116_Supp_Materials.tar.gz › ESAT-esat_v0.1/doc/html/classbroad_1_1core_1_1hmm_1_1_markov_model_3_01_t_01_4.html]

ESAT: broad.core.hmm.MarkovModel< T > Class Reference


ESAT


- Main Page
- Related Pages
- Packages
- Classes
- Files

- Class List
- Class Index
- Class Hierarchy
- Class Members

All Classes Namespaces Files Functions Variables Enumerator Pages

- broad
- core
- hmm
- MarkovModel< T >

Classes |
Public Member Functions |
Protected Member Functions |
List of all members

broad.core.hmm.MarkovModel< T > Class Reference

|  |  |
| --- | --- |
| Classes | |
| class | **BackwardResult< T >** |
|  | |
| class | **ForwardResult< T >** |
|  | |

|  |  |
| --- | --- |
| Public Member Functions | |
|  | MarkovModel (int numberOfStates) |
|  | |
| void | addState (MarkovState< T > state) |
|  | |
| void | addState (MarkovState< T > state, int stateIdx) |
|  | |
| void | setInitialStateTransitionProbability (int stateIdx, double probability) |
|  | |
| void | setEndStateTransitionProbability (int stateIdx, double probability) |
|  | |
| MarkovState< T > | getState (String stateName) |
|  | |
| List< Integer > | emitPath (int numOfSteps) |
|  | |
| void | setStateTransitionProbability (int stateIdx1, int stateIdx2, double probability) |
|  | |
| double | computePathLogLikelihood (List< T > observationSequence, short[] path, int initialStateIdx, int endStateIdx) |
|  | |
| short[] | viterbiMostLikelyEstimation (List< T > observationSequence) throws BadModelException |
|  | |
| ForwardResult< T > | runForwardAlgorithm (List< T > observationSequence) throws BadModelException |
|  | |
| BackwardResult< T > | runBackwardAlgorithm (List< T > observationSequence, ForwardResult< T > forwardData) throws BadModelException |
|  | |
| ForwardResult< T > | runForwardAlgorithmNoRescaling (List< T > observationSequence) |
|  | |

|  |  |
| --- | --- |
| Protected Member Functions | |
| Matrix | getStateTransitionMatrix () |
|  | |
| List< MarkovState< T > > | getStates () |
|  | |
| double[] | getInitialStateTransitionProbabilities () |
|  | |
| List< String > | getConsistencyProblems () |
|  | |
| int | drawInitialState () |
|  | |
| int | drawNextState (int prior) |
|  | |

## Constructor & Destructor Documentation

|  |  |  |  |  |  |
| --- | --- | --- | --- | --- | --- |
| broad.core.hmm.MarkovModel< T >.MarkovModel | ( | int | *numberOfStates* | ) |  |

Initialize the Model by specifying the number of states. The probabilities of starting with a given state will be initialized as uniform: All states are equally likely at start

Parameters
:   |  |  |
    | --- | --- |
    | numberOfStates |  |

## Member Function Documentation

|  |  |  |  |  |  |
| --- | --- | --- | --- | --- | --- |
| void broad.core.hmm.MarkovModel< T >.addState | ( | MarkovState< T > | *state* | ) |  |

|  |  |  |  |
| --- | --- | --- | --- |
| void broad.core.hmm.MarkovModel< T >.addState | ( | MarkovState< T > | *state*, |
|  |  | int | *stateIdx* |
|  | ) |  |  |

|  |  |  |  |
| --- | --- | --- | --- |
| double broad.core.hmm.MarkovModel< T >.computePathLogLikelihood | ( | List< T > | *observationSequence*, |
|  |  | short[] | *path*, |
|  |  | int | *initialStateIdx*, |
|  |  | int | *endStateIdx* |
|  | ) |  |  |

Here is the call graph for this function:

|  |  |  |  |  |
| --- | --- | --- | --- | --- |
| int broad.core.hmm.MarkovModel< T >.drawInitialState | ( |  | ) |  |

protected

|  |  |  |  |  |  |
| --- | --- | --- | --- | --- | --- |
| int broad.core.hmm.MarkovModel< T >.drawNextState | ( | int | *prior* | ) |  |

protected

|  |  |  |  |  |  |
| --- | --- | --- | --- | --- | --- |
| List<Integer> broad.core.hmm.MarkovModel< T >.emitPath | ( | int | *numOfSteps* | ) |  |

|  |  |  |  |  |
| --- | --- | --- | --- | --- |
| List<String> broad.core.hmm.MarkovModel< T >.getConsistencyProblems | ( |  | ) |  |

protected

|  |  |  |  |  |
| --- | --- | --- | --- | --- |
| double [] broad.core.hmm.MarkovModel< T >.getInitialStateTransitionProbabilities | ( |  | ) |  |

protected

|  |  |  |  |  |  |
| --- | --- | --- | --- | --- | --- |
| MarkovState<T> broad.core.hmm.MarkovModel< T >.getState | ( | String | *stateName* | ) |  |

Here is the call graph for this function:

|  |  |  |  |  |
| --- | --- | --- | --- | --- |
| List<MarkovState<T> > broad.core.hmm.MarkovModel< T >.getStates | ( |  | ) |  |

protected

|  |  |  |  |  |
| --- | --- | --- | --- | --- |
| Matrix broad.core.hmm.MarkovModel< T >.getStateTransitionMatrix | ( |  | ) |  |

protected

|  |  |  |  |
| --- | --- | --- | --- |
| BackwardResult<T> broad.core.hmm.MarkovModel< T >.runBackwardAlgorithm | ( | List< T > | *observationSequence*, |
|  |  | ForwardResult< T > | *forwardData* |
|  | ) |  | throws BadModelException |

|  |  |  |  |  |  |
| --- | --- | --- | --- | --- | --- |
| ForwardResult<T> broad.core.hmm.MarkovModel< T >.runForwardAlgorithm | ( | List< T > | *observationSequence* | ) | throws BadModelException |

|  |  |  |  |  |  |
| --- | --- | --- | --- | --- | --- |
| ForwardResult<T> broad.core.hmm.MarkovModel< T >.runForwardAlgorithmNoRescaling | ( | List< T > | *observationSequence* | ) |  |

|  |  |  |  |
| --- | --- | --- | --- |
| void broad.core.hmm.MarkovModel< T >.setEndStateTransitionProbability | ( | int | *stateIdx*, |
|  |  | double | *probability* |
|  | ) |  |  |

|  |  |  |  |
| --- | --- | --- | --- |
| void broad.core.hmm.MarkovModel< T >.setInitialStateTransitionProbability | ( | int | *stateIdx*, |
|  |  | double | *probability* |
|  | ) |  |  |

|  |  |  |  |
| --- | --- | --- | --- |
| void broad.core.hmm.MarkovModel< T >.setStateTransitionProbability | ( | int | *stateIdx1*, |
|  |  | int | *stateIdx2*, |
|  |  | double | *probability* |
|  | ) |  |  |

|  |  |  |  |  |  |
| --- | --- | --- | --- | --- | --- |
| short [] broad.core.hmm.MarkovModel< T >.viterbiMostLikelyEstimation | ( | List< T > | *observationSequence* | ) | throws BadModelException |

---

The documentation for this class was generated from the following file:

- java/broad/core/hmm/MarkovModel.java


---

Generated on Fri Aug 15 2014 15:10:52 for ESAT by  

 1.8.7
